# Development and validation of the caregiver roles and responsibilities scale in cancer caregivers

**DOI:** 10.1007/s11136-019-02154-4

**Published:** 2019-03-18

**Authors:** Valerie Shilling, Rachel Starkings, Valerie Jenkins, David Cella, Lesley Fallowfield

**Affiliations:** 1 Sussex Health Outcomes Research and Education in Cancer (SHORE-C), Brighton and Sussex Medical School, University of Sussex, Brighton, UK; 2 Department of Medical Social Sciences, Northwestern University Feinberg School of Medicine, Chicago, IL USA

**Keywords:** Caregiving, Cancer, Outcome measures, Psychometric performance, Validation, Questionnaire development

## Abstract

**Purpose:**

The caregiver roles and responsibilities scale (CRRS) was developed to facilitate formal assessment of broad life impacts for informal (i.e. unpaid) caregivers to people with cancer. Here we report the development and initial validation.

**Methods:**

The CRRS was developed from the thematic analysis of two interview studies with cancer patients (stage III-IV breast, gynaecological, lung or melanoma) and caregivers. In the evaluation studies, participants completed the CRRS alongside the Caregiver Quality of Life—Cancer, the main criterion measure for concurrent validity, and the WHOQOL-BREF for additional convergent validity data. Questionnaires were completed at baseline, 7-days and 2-months. Demographic data and patient characteristics were collected at baseline.

**Results:**

Two-hundred and forty-five caregivers to people with stage I-IV breast, colorectal, gynaecological, head and neck, lung or renal cancer or melanoma completed the CRRS at least once. The final 41 core items selected comprised five subscales: Support and Impact, Lifestyle, Emotional Health and Wellbeing, Self-care and Financial Wellbeing as well as three standalone items. Missing data rate was low (0.6%); there were no ceiling or floor effects for total scores. Cronbach’s alpha was 0.92 for the CRRS-41; 0.75–0.87 for the subscales. CRRS showed good test–retest reliability (ICC = 0.91), sensitivity to change and the predicted pattern of correlation with validation measures *r* = 0.75–0.89. The standalone 7-item jobs and careers subscale requires further validation.

**Conclusions:**

Initial evaluation shows the CRRS has good validity and reliability and is a promising tool for the assessment of the effects of cancer and cancer treatment on the lives and wellbeing of informal caregivers.

**Electronic supplementary material:**

The online version of this article (10.1007/s11136-019-02154-4) contains supplementary material, which is available to authorized users.

## Background

Informal (i.e. unpaid) caregivers of patients, such as spouses, family members or friends, frequently provide significant amounts of care and support [1, 2] often alongside their many other responsibilities [3–6]. Informal caregiving contributes to patients’ overall experiences of treatment, therefore maintaining their wellbeing and satisfaction is essential [1, 2].

Caregivers must manage several factors relating directly to a patient’s illness together with other ‘secondary stressors’ [4] such as changing roles, disruption to lifestyle and family functioning, changes to relationships and sense of self, concerns regarding their own and the patient’s employment with the associated financial implications [4]. Additionally informal caregivers may also be caring for children and/or other family members, which can complicate and compound the more negative aspects and effects of being a primary caregiver to someone with cancer [3, 7, 8]. Informal caregivers play a crucial role, yet formal assessment of the effects on their lives and wellbeing is not routine.

Our systematic review [9] revealed that few of the measures used for caregiver quality of life and caregiver burden were developed and validated specifically in the cancer caregiver population. In addition, most were developed a long time ago, when the advances in medicine that now allow so many patients to live with cancer as a chronic condition, and the associated adaptations required to their own and their family’s lives, had not been made. We found notable gaps in item coverage in a concept mapping exercise to examine the content of these measures. Results suggested that currently available scales do not capture adequately changes in occupational, financial, household and family roles and responsibilities as a result of caregiving, or the broader impacts on the family unit. The influence on career aspiration and progression were not addressed at all; for example, the opportunity cost of a missed promotion at work [9]. Caregivers experience a sense that their own life is suspended, ‘on hold’ in some way; that the uncertainty around the length and quality of the patient's survival affects wide-ranging areas of life [10–13]. It is vital that this lack of control and altered sense of agency is better captured in caregiver outcome measures.

Our systematic reviews of Patient Reported Outcome Measures in use in the cancer patient [14] and cancer caregiver [9] populations highlighted the need for new rigorously developed, well-validated quality of life measures to assess these broader impacts of disease and treatment.

## Overview

The PROACT (Patient Reported Outcomes, impact of Age and Carer role demands associated with Treatment) programme of work is a multi-phase project, informed by the Quality of Survival (QoS) concept framework [15]. The measures were developed to be used alongside the Functional Assessment of Chronic Illness Therapy (FACIT) measurement system and are intended to capture the broader impact of cancer and its treatment during the evaluation of new treatments in clinical trials. Results from questionnaire completion might also enable discussions between clinicians, patients and caregivers about potential treatments and supportive interventions.

Previously we described the development and initial validation of the Patient Roles and Responsibilities Scale (PRRS) [16]. This paper reports the four studies that comprised the development and evaluation of the Caregiver Roles and Responsibilities Scale (CRRS). The initial development studies were conducted with caregivers to people with stage III-IV cancer. This was to reflect the fact that due to scientific advances, there has been a large increase in the number of patients living with their cancer, rather than being cured of it, while trying to maintain ‘normal’ life including family, financial and employment responsibilities. These aspects of life are significantly impacted for caregivers as well as patients and we wanted to ensure the measure captured these. The measure was, however, developed with the intention to be appropriate for caregivers to patients with any stage or type of cancer and the later validation study includes caregivers to patients with stage I-II cancer.

## Ethics statement

Studies 1–3 received ethics approval from London Queen Square Research Ethics Committee (ref: 15/LO/1323; 14th September 2015 and ref: 16/LO/1125; 6th September 2016). Study 4 received ethics approval from London Central Research Ethics Committee (ref: 17/LO/1773; 12th October 2017). Signed informed consent was obtained from all participants.

### Eligibility criteria

Inclusion/exclusion criteria were broadly the same across all four studies. Eligible patients had advanced (stage III/IV) lung cancer, gynaecological cancer or melanoma. For studies 1 and 2, we required that patients nominate an informal caregiver also willing to take part. Both cohorts were required to be over 18 years of age, have capacity to give informed consent and be able to read and speak in English (essential for the purposes of scale development and evaluation). Individuals who were inpatients or acutely distressed for any reason were excluded. In studies 2 and 3 we expanded the patient population to include women with breast cancer. In study 3 we relaxed the requirement that both patient and caregiver consent to the study; either party could participate alone. In study 4 we expanded the population further to include patients with renal, head and neck and colorectal cancer and those whose cancer was stage I/II. This is because the questionnaire has been developed to be suitable for use by people supporting patients with cancer of all types and stages.

## Scale development: studies 1 and 2

### Study 1: item generation

We conducted in depth qualitative interviews with 24 patients with advanced cancer and their nominated informal caregivers (*N* = 23) about the influence of extended cancer survival on broader aspects of life and wellbeing (see supplementary file S1 for participant demographics). Interviews were recorded and transcribed verbatim.

A thematic framework was developed from an initial process of open coding and tested iteratively as new data were collected. Thematic analysis identified 20 themes and 33 sub-themes from which a list of 181 potential items was generated for the caregiver scale. These were reviewed for relevance and redundancy by the authors before 51 potential items were viewed by our panel of advisors. 64 items were retained for evaluation in Study 2. The Caregivers Roles and Responsibilities Scale will sit within the FACIT measurement system. As such, when concepts were identified that were important to caregivers, we checked if existing items within the FACIT library were appropriate rather than generating new items; those items being already validated measures of the concept. It would be unnecessary and even inappropriate to generate new items on the same content when fully validated (and often translated into multiple languages) items exist within the measurement system that the CRRS will be part of. With permission, we included 3 items from the FACIT Comprehensive Score for Financial Toxicity (COST) measure [17], 3 items from the Functional Assessment of Cancer Therapy-General (FACT-G) [18] and 1 item from FACIT-Spirituality (FACIT-Sp) [19]. For further detail on the procedure and analysis of these interviews, see the related open access paper [16].

### Study 2: item reduction and scale construction

Cognitive interviews were conducted with a new cohort of 20 patients and their informal caregivers using a mixture of the ‘think aloud’ technique and specific probes around comprehension, retrieval, judgement and response options to assess each of the potential scale items. Items were also discussed in terms of acceptability, relevance, redundancy and importance. Participants were also given the opportunity to identify missing topics. See supplementary file S1 for participant demographics.

Scale items were revised, added and removed in an iterative fashion through the course of the study. Ninety-four changes were made to the caregiver scale including 80 wording revisions, 9 deletions and 5 additions resulting in a 60 item scale. In order to present the questionnaire in manageable ‘chunks’ for the participant we loosely grouped items under the headings: Family and Support (*N* = 12); Relationships and Communication (*N* = 7); Lifestyle and Outlook (*N* = 12); Health and Wellbeing (*N* = 14); Financial Wellbeing (*N* = 6) and Jobs and Careers (*N* = 9). We did not theorise that these groupings necessarily reflect the underlying factor structure.

### Evaluation and validation: studies 3 and 4

Studies 3 and 4 were conducted consecutively. In study 3, participants completed the full 60 item CRRS as described below. CRRS individual item data were then analysed for the purpose of item reduction. Participants in study 4 completed the reduced CRRS. Data from the reduced CRRS from both studies were analysed together for the Exploratory Factor Analysis, test retest reliability, criterion and convergent validity and sensitivity to change.

## Methods

### Population and procedure

Study 3 participants were recruited from 11 sites in England. Participants were the informal caregivers to patients with stage III/IV breast, gynaecological or lung cancer or malignant melanoma. Study 4 participants were recruited from 13 sites in England. Participants were the informal caregivers to patients with stage I-IV breast, colorectal, gynaecological, head and neck, lung or renal cancer or malignant melanoma.

Participants completed questionnaires at home either on paper or online, whichever their preference. Participants completing online were not forced to respond to all items, although an onscreen message notified them of omissions. Where participants missed multiple items, whether completing on paper or online, they were followed up by the study team to determine whether the omissions were intentional or if they had skipped them accidentally (with the option to provide a response at this point). Demographics and the full validation pack were completed at baseline, the CRRS was completed alone after 7 days (for test–retest) and the full pack completed again after 2 months (sensitivity to change).

### Measures

Participants completed the CRRS alongside a validation battery including the WHOQOL-BREF [20] and the Caregiver Quality of Life—Cancer [21]. They provided basic demographic information such as age, employment status, level of education, relationship status, caregiving responsibilities and information regarding the diagnosis and treatment of the person they support.

#### The CRRS

The CRRS as completed in Study 3 comprised 60 items: 51 Core Items formatted for the FACIT measurement system (responses as item applies to the past 7 days, 5 response options ranging from Not at All—Very Much) and 1 binary response item on whether the participant has stopped work due to their caregiving responsibilities. A standalone scale, Jobs and Careers comprising 2 binary response items and 7 Likert-scale items is completed only by participants currently in paid employment. Negatively worded items were reverse scored so that a higher score corresponds to better Quality of Life. Participants in Study 4 completed the reduced CRRS with 41 Core Items and the Jobs and Careers subscale.

#### The WHOQOL-BREF [20]

The WHOQOL-BREF is a 26 item scale that produces a quality of life profile with four domain scores (physical health, psychological health, social relationships and environment) and two individually scored items about an individual’s overall perception of quality of life and health. Each item is rated on a 5 point scale (scored 1–5). The four domain scores are scaled in a positive direction with higher scores indicating a higher quality of life.

#### The caregiver quality of life cancer scale (CQOLC) [21]

The CQOLC is a well-validated and extensively used quality of life measure developed specifically for caregivers of people with cancer. It has 35 items rated on a five point scale (not at all—very much). Scores range from 0 to 140 with higher scores indicating higher quality of life. It comprises domains relating to burden, disruptiveness, finances and positive adaptation. Our recent systematic review [22] found the CQOLC, alongside the Caregiver Reaction Assessment [23] had the strongest support for its psychometric performance.

### Data analysis

Analyses were conducted using the Statistical Package for Social Sciences (IBM SPSS; version25). Missing data from CRRS, although rare, were managed by prorating the total for selected analyses. Missing data from other questionnaires were managed as per the specific instrument guidance. We used guidelines from the International Society for Pharmacoeconomics and Outcomes Research (ISPOR) [24, 25] and COSMIN (Consensus-based Standards for the Selection of Health Measurement Instruments) [26] in the development and evaluation of this measure.

On the completion of Study 3, the CRRS was assessed in terms of acceptability (missing data, time to complete) and precision (floor/ceiling effects, skewness, inter-item correlations) and a number of items removed.

Combined data from Study 3 and 4 were then analysed using Exploratory Factor Analysis (PCA with oblique rotation); internal consistency (Cronbach’s alpha); criterion and convergent validity (correlation); test–retest reliability (Intraclass Correlation, two-way random, absolute agreement; weighted Kappa for individual items) and responsiveness to change (caregivers were categorised as improved, worsened or unchanged based on meaningful change in WHOQOL-BREF. Paired t-tests were used to determine if CRRS change within a group was significantly different from zero. Spearman correlation coefficients were calculated to explore the relationship between change on PRRS and change on WHOQOL-BREF and CQOLC). Data analysis methods, including thresholds, are explained in greater detail in the related open access paper [16].

## Results study 3

### Participants

Of the 119 caregivers who consented to take part, 110 completed baseline assessment (29 caregivers to patients with breast cancer, 31 gynaecological, 31 lung and 19 melanoma), 103 completed T2 and 96 completed T3. At baseline, age ranged from 18 to 88 (median 60 years); 57% were male. Relationship to patient was: partner/spouse (*N* = 83, 75%); child of patient (*N* = 14, 13%); friend (*N* = 5, 4%); sibling (*N* = 4, 4%) parent of patient (*N* = 2, 2%) and ‘other’ (*N* = 2, 2%).

### Acceptability and precision of CRRS

Missing data rates for the CRRS were extremely low; 0.3% at baseline, 0.1% at Time 2 and 0.1% at Time 3. Missing data were distributed across 11 of the 58 Likert-scale items at baseline, 8 items at Time 2 and 5 at Time 3 with no single item having more than 2 missing responses at any time point. We had set a threshold that items with > 15% missing data would be investigated for acceptability. The highest rate of missing data for a single item at any time point was 4% to an employment question with 45 responses of a possible 47; for core questions with the larger sample, the highest rate of missing data for a single item at any time point was 2%.

At baseline, 62 participants completed questionnaires online, 48 completed on paper, but only 43 recorded the time taken to complete the CRRS. Time to complete online ranged from 2.9 to 20.6 min with 1 extreme outlier at 34.4 min (mean excluding outlier = 9.3 min, SD = 4.0 min). On paper, time to complete ranged from 8 to 25 min, (excluding outliers above the 90th percentile), mean = 13.2 min, SD = 4.5 min.

### CRRS core items: item reduction

None of the individual items had missing data > 15%. Scores of minimum ‘0’ ranged from 0.9 to 90.9%; on 42/51 items less than 20% of responses were the minimum ‘0’. 1 item had floor effects exceeding 70% (90.9%—‘I take part in support groups and/or internet forums’).

Four items showed above threshold ceiling effects (72.7% ‘My caregiving responsibilities are a burden’ [reverse scored]; 77.8% ‘I feel appreciated by X’; 79.1% ‘I have difficulty meeting the additional costs of supporting X’ [reverse scored]; 83.6% ‘I feel close to X’). Scores of maximum ‘4’ ranged from 0.9 to 83.6%; 15/51 items had 20% or less responses at maximum ‘4.

Eleven items had an unacceptably low level of correlation with other items and low (< 0.3) corrected item-total correlations in reliability analysis and concerns around face validity. In addition five of these did not meet the measure of sampling adequacy (set at 0.7). Ten of the eleven were removed from further analysis including the item with above threshold floor effects and 2 of the 4 items with above threshold ceiling effects. The item Sp9 was retained because it was felt important to keep an item pertaining to spiritual wellbeing.

Of the 41 retained Likert-scale core items, 14 had z-score of skewness > ǀ4ǀ however their performance in terms of relationship to other items, corrected item-total correlation and/or conceptual significance to the measure ensured their retention at this stage. Total scores on the modified CRRS Core-41 (*N* = 110) ranged from 33 to 148 (possible range 0-164), mean = 105.56, SD = 23.48 with skewness = − 0.73(SE = 0.23).

## Results study 3 and 4

### Participants

**Fig. 1:**
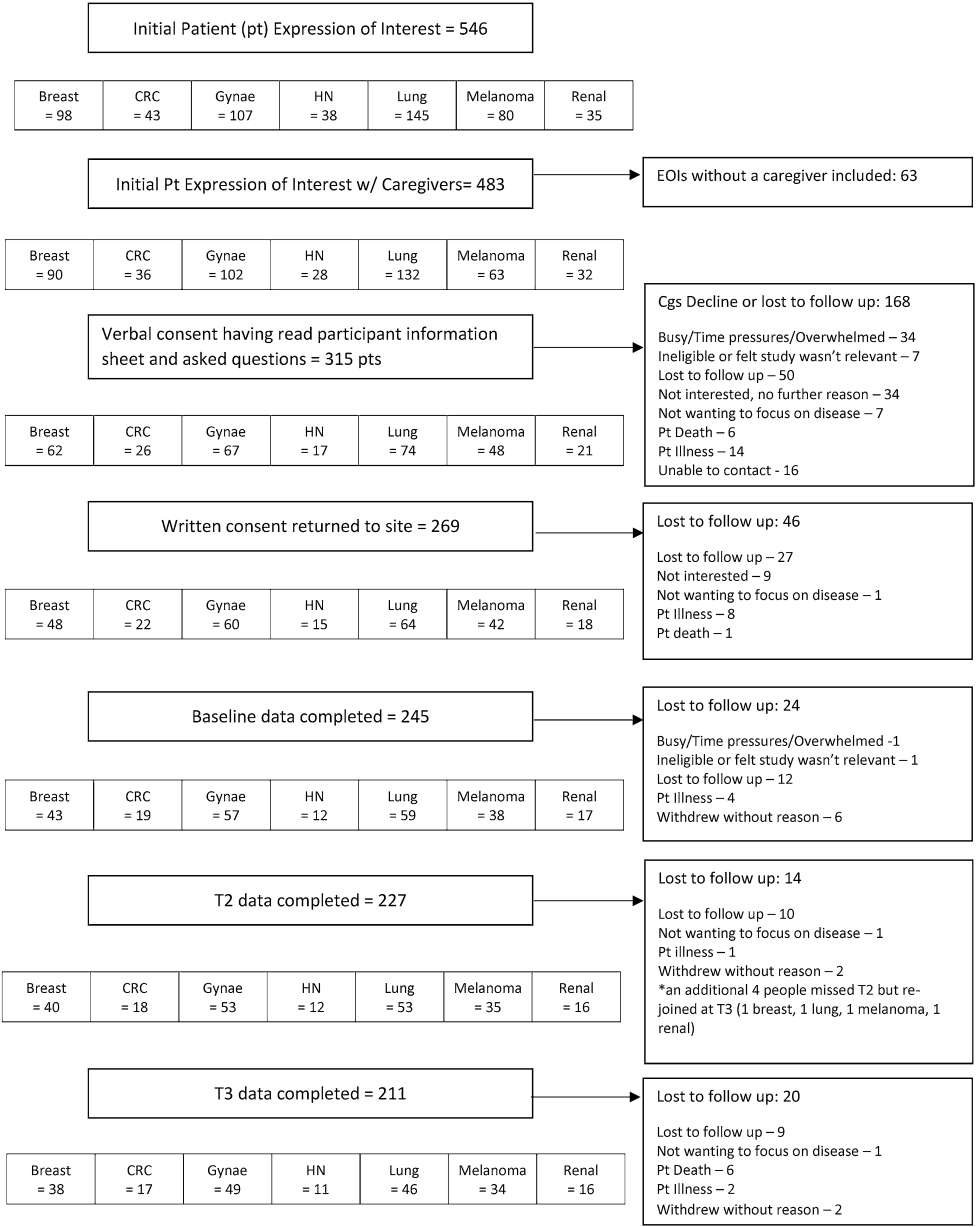
Flow of participants through study

Of the 269 caregivers who consented to take part, 245 completed baseline assessment (59 caregivers to patients with lung cancer, 57 gynaecological, 43 breast, 38 melanoma, 19 colorectal, 17 renal and 12 head and neck), 227 completed T2 and 211 completed T3. At baseline, age ranged from 18 to 89 (median 60 years); 52% were male. Relationship to patient was: partner/spouse (*N* = 192, 78%); child of patient (*N* = 22, 9%); friend (*N* = 12, 5%); sibling (*N* = 8, 3%) parent of patient (*N* = 8, 3%) and ‘other’ (*N* = 3, 1%). See Fig. 1 for full details of caregivers approached, consented and completed questionnaires at each time point, along with reasons for decline/drop out and Table 1 for key participant characteristics.

**Table 1:**
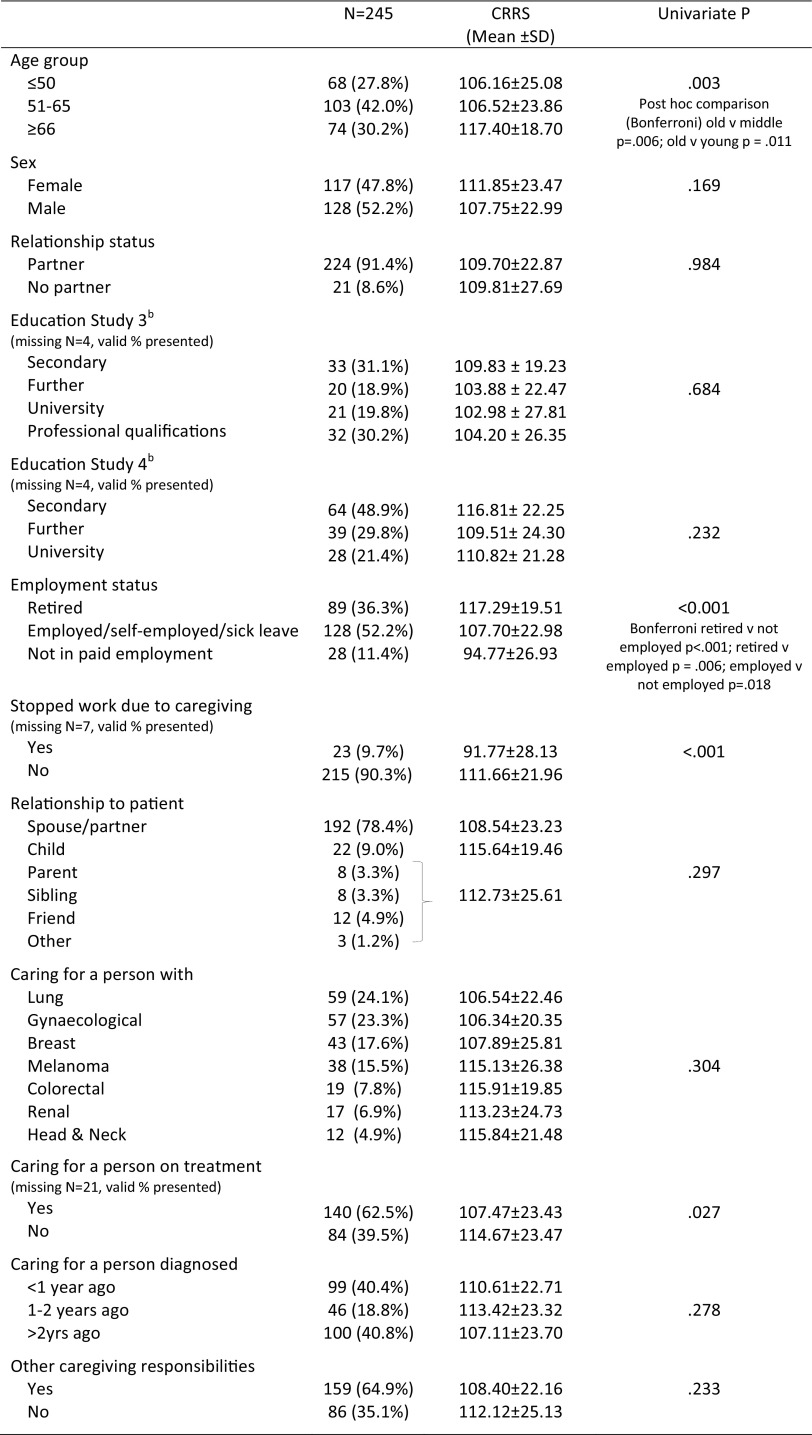
Key caregiver characteristics and CRRS-41 scores

^a^Ethnicity is not reported as 94% of participants identified as White British

^b^The question relating to education was posed differently in study 3 (highest level of education, one choice only including professional qualifications) and study 4 (education and professional/ vocational qualifications reported separately)

Missing data rates for the CRRS were very low: 0.6% at baseline; 0.4% at Time 2 and 0.4% at Time 3. Missing data were distributed across 27 of the 48 Likert-scale items at baseline; in the majority of cases with only one or two missing responses from 245 participants. Three items in particular had higher missing data rates of 10 (4%), 8 (3%) and 5 (2%). Missing data at T2 were distributed across 22/48 items. Two of the same items again had higher rates of missing data with 8 (4%) and 6 (3%) missing responses. At T3 missing data were distributed across 22/48 items.

We had set a threshold that items with > 15% missing data would be investigated for acceptability. While none of the items had missing data close to this level, the three items identified also showed a very low pattern of correlation with other items (*r* < 0.3) and low corrected item-total correlations in reliability analysis. As such, they were not included in the exploratory factor analysis and do not sit within the subscales identified therein. They do however cover important aspects of wellbeing not measured by other items in the scale and are retained as standalone items (see Table 2 for details).

**Table 2:** Standalone items

Item	Inter-item correlation range	Median/mode	$\left| {{\text{z-score}}\;{\text{skew}}} \right|$	% Floor (score 0)	% Ceiling (score 4)	Weighted κ
I am less able to fulfil my other caregiving responsibilities (e.g. looking after children, grandchildren, another adult, pets)	0.01–0.29	4/4	10.16	2	63	0.43
I worry about the impact of X’s illness on my children and/or other family members	0.008–0.31	1/1	1.86	24	9	0.56
I find comfort in my faith or spiritual beliefs	0.002–0.24	0/0	10.27	63	7	0.75

### Exploratory factor analysis

Thirty-eight items were entered into the EFA. Following listwise deletion based on all variables, *N* = 228 (COSMIN guidelines [26] consider an item to participant ratio of 1:5 to be ‘good’; this ratio is 1:6). Bartlett’s test of sphericity was significant at < 0.0001 and the Kaiser–Meyer–Olkin (KMO) value was 0.87, confirming that the data were suitable for factor analysis. All items had above threshold (> 0.7) MSA suggesting adequate communality with other variables.

Nine eigenvalues were greater than 1.0, explaining a total of 64.3% of variance. Consideration of the scree plot suggested that 5 factors might be retained explaining 26.9, 8.8, 6.1, 5.2 and 4.8% of variance respectively (51.8% total variance). Items and their factor loadings are shown in Table 3. Most (33/38) items loaded clearly on one factor; the remaining had loadings < 0.4 on all factors (range 0.33–0.39).

**Table 3:** Item factor loadings

	Item	Factor 1 Emotional health and wellbeing	Factor 2 Support and impact	Factor 3 Lifestyle	Factor 4 Financial wellbeing	Factor 5 Self-care
CH8	I feel depressed by the situation	0.77				
GE1	I feel sad	0.77				
CH6	I feel angry about the situation	0.73				
CO8	I find it hard to think about my own future	0.73				
CO10	I feel angry that our plans for the future have changed because of X’s illness	0.71				
CO6	It is difficult to think about anything other than X’s illness	0.69				
CH7	I feel overwhelmed by the situation	0.66				
CH11	I feel resentful that my life has changed because of X’s illness	0.50				
CR3	I find some of X’s decisions about treatment difficult to accept	*0.38*				
CS56	Friends and family provide emotional support		0.86			
CS22	Friends and family provide practical support		0.81			
CS24	People are interested in how I am coping		0.74			
CS55	My family and I support each other		0.71			
CS21	I feel the cancer team recognise the impact on me		0.54			
CS53	I feel that support is available from the health system		0.53			
CO1	I keep to my normal routines and activities			0.76		
CO2	I socialise less because of my caregiving responsibilities			0.74		
CS2	I have taken on some of X’s responsibilities at home (e.g. cooking, cleaning, gardening, DIY)			0.72		
GF6	I am enjoying the things I usually do for fun			0.62		
CO4	I make time to do things for myself			0.58		
CH4	My physical health has suffered as a result of the situation			0.53		
CO7	My caregiving responsibilities are a burden			0.52		
CO9	I feel as if my life is on hold			0.50		
GF7	I am content with the quality of my life right now			0.42		
CO5	I feel guilty taking time for myself			*0.34*		
CH5	My mental health has suffered as a result of the situation			*0.33*		
CF4	I have difficulty meeting the additional costs of supporting X				0.66	
FT11	I feel in control of my financial situation				0.61	
FT3	I worry about the financial problems I will have in the future as a result of X’s illness or treatment				0.61	
CF3	The additional costs of supporting X are more than I thought they would be (e.g. travel and parking, heating, healthy eating, supplements, non-prescription medication, paying for help at home)				0.55	
CF2	I have to help X financially as a result of his/her illness				0.50	
CF1	I know where to access financial advice and support				0.41	
CH9	I feel confident in my ability to support X					0.63
CR4	X and I speak openly with each other about his/her illness					0.60
CH3	I look after myself emotionally					0.51
CR6	I protect myself by not talking about X’s illness					0.42
CH1	I have a positive attitude to life					*0.39*
CH2	I look after myself physically^a^			*0.37*		*0.37*

Factor loadings < 0.4 are suppressed, unless an item does not load at > 0.4 on any factor. The highest factor loading is then shown in italics

^a^The item CH2 loads equally on Factors 5 and 3. This item has been retained in Factor 5 (and the associated subscale *self-care*) as this is where it has most face validity

The five factors were labelled and evaluated as potential subscales: Emotional Health and Wellbeing (Cronbach’s α = 0.87, corrected item-total correlation range 0.33 to 0.72); Lifestyle (Cronbach’s α = 0.87, corrected item-total correlation range 0.49 to 0.70); Support and Impact (Cronbach’s α = 0.82, corrected item-total correlation range 0.52 to 0.69); Self-care (Cronbach’s α = 0.75, corrected item-total correlation range 0.36 to 0.68) and Financial Wellbeing (Cronbach’s α = 0.78, corrected item-total correlation range 0.43 to 0.68) Inter-item and inter-subscale correlations are shown in Table 4.

**Table 4:** Inter-item correlations and correlation between subscale scores

Subscale	Mean (SD) Range	Inter-item correlation range	Correlations between subscale scores				
			Emotional health and wellbeing	Lifestyle	Support and impact	Self-care	Financial wellbeing
Emotional Health and Wellbeing (9 items, possible score 0–36)	25.14 (7.65) 3–36 *N* = 241	0.14 to 0.65 29/36 *r* > 0.3 *N* = 241	1.00				
Lifestyle (11 items, possible score 0–44)	29.03 (8.49) 5–44 *N* = 237	0.22 to 0.60 45/55 *r* > 0.3 *N* = 237	0.62 *N* = 235	1.00			
Support and Impact (6 items, possible score 0–24)	13.65 (5.47) 2–24 *N* = 237	0.22 to 0.65 12/15 *r* > 0.3 *N* = 237	0.17 *N* = 235	0.19 *N* = 230	1.00		
Self-care (6 items, possible score 0–24)	18.02 (3.98) 4–24 *N* = 243	0.14 to 0.68 6/15 *r* > 0.3 *N* = 243	0.52 *N* = 240	0.48 *N* = 237	0.35 *N* = 235	1.00	
Financial Wellbeing (6 items, possible score 0–24)	17.76 (5.02) 4–24 *N* = 242	0.12 to 0.59 10/15 *r* > 0.3 *N* = 242	0.50 *N* = 240	0.52 *N* = 235	0.22 *N* = 236	0.36 *N* = 240	1.00

### Internal consistency

Cronbach’s α for the CRRS full scale at baseline was 0.92 (corrected item-total correlation range 0.09–0.65); at T2 α = 0.94 (CITC range 0.10–0.71) and at T3 α = 0.93 (CITC range 0.12–0.71). If the three standalone items are excluded, α at baseline was 0.92 (corrected item-total correlation range 0.24–0.64); at T2 α = 0.94 (CITC range 0.32–0.71) and at T3 α = 0.93 (CITC range 0.25–0.71).

### Criterion and convergent validity

Total scores for participants with full data sets on the CRRS Core-41 (*N* = 219) ranged from 33 to 152 (possible range 0–164), mean = 108.74, SD = 23.07 with skewness = − 0.58 (SE = 0.16). As predicted, total scores on the CRRS correlated strongly with scores on the gold standard measure, the Caregiver Quality of Life—Cancer (*r* = 0.89, *N* = 217) and the other validation measure the WHOQOL-BREF (*r* = 0.75, *N* = 214).

Removing the three standalone items from the total score results in a range from 32 to 148 (possible range 0–152), *N* = 228, mean = 103.04, SD = 22.39 with skewness = − 0.60 (SE = 0.16) correlating with CQOLC (*r* = 0.88, N = 226) and WHOQOL-BREF (*r* = 0.74, *N* = 223).

### Test–retest reliability

The median number of days between baseline and T2 was 9 (range 4–26). Two participants were excluded from analysis as T2 was completed too far from baseline. Total CRRS scores showed good test–retest reliability (two-way random, absolute agreement) (ICC = 0.91, 95% CI 0.88–0.93) as did the five potential subscales: Emotional health and wellbeing (ICC = 0.85, 95% CI 0.81–0.89); Lifestyle (ICC = 0.86, 95% CI 0.82–0.89); Support and impact (ICC = 0.77, 95% CI 0.71–0.82); Self-care (ICC = 0.79, 95% CI 0.73–0.84) and Financial Wellbeing (ICC = 0.88, 95% CI 0.85–0.91).

Weighted kappa for individual items were all in the acceptable range (κ ≥ 0.4) excepting one at κ = 0.39 (I find some of X’s decisions about treatment difficult to accept). Kappa ranged from 0.39 to 0.75 with 31 items in the ‘acceptable’ range (0.4 ≤ κ ≤ 0.59) and 9 in the ‘good’ range (κ ≥ 0.6).

### Responsiveness to change

We have calculated responsiveness to change using two methods. First, we have prorated total CRSS scores for those participants who missed one item or more at baseline or T3. This maximises the number of participants included in the analysis and prorating, if anything, will dull responsiveness to change. As some experts may query prorating in a validation study, we have also analysed the data excluding those with any missing item at either time point. The median number of days between baseline and T3 was 66 (range 56–103).

Using prorated total CRRS scores, *N* = 208. Participants were categorised as improved, worsened or unchanged on WHOQOL-BREF using an effect size of 0.2 as a minimum threshold for significant change. Twenty-six percent were categorised as ‘unchanged’, 25% as ‘improved’ and 49% as ‘worsened’. Participants who showed meaningful decline on WHOQOL-BREF also showed significant decline on CRRS scores (*p* < 0.001). Those who improved showed significant improvement on CRRS (*p* = 0.001). CRRS scores did not change significantly for those categorised as unchanged on WHOQOL-BREF (*p* = 0.196). Using a more conservative effect size of 0.5 as a threshold for change, 65.5% were categorised as ‘unchanged’, 26.5% as ‘worsened’ and 8% as ‘improved’. Participants categorised as worsened showed significant decline on CRRS scores (*p* < 0.001). Those categorised as improved showed significant improvement on CRRS (*p* = 0.001). CRRS scores did not change significantly for participants categorised as unchanged (*p* = 0.288).

Including only participants who had answered all items on the scale, *N* = 171. The pattern of results was very similar using the 0.2 effect size for change on WHOQOL-BREF: 25% were categorised as unchanged; 26% improved and 49% worsened with those showing meaningful decline also showing significant decline on CRRS scores (*p* < 0.001), those who improved showed significant improvement on CRRS (*p* = 0.003) and no significant change in CRRS scores for those categorised as unchanged on WHOQOL-BREF (*p* = 0.371). Using the effect size of 0.5 as a threshold for change, 64% were categorised as ‘unchanged’, 27% as ‘worsened’ and 9% as ‘improved’. As before those who showed meaningful decline or improvement also showed significant decline or improvement on the CRRS (*p* < 0.001 and *p* = 0.003 respectively). CRRS scores did not change significantly for those categorised as unchanged (*p* = 0.496). Table 5 shows mean CRRS change by group for both analyses.

**Table 5:** CRRS change by change on WHOQOL-BREF

WHOQOL-BREF	N	Baseline CRRS mean (SD)	Mean CRRS change (SD)	*p*	Effect size^a^
Using prorated CRRS scores if missing data present *N* = 208					
0.2 effect size					
Improved	52	108.60 (25.00)	− 5.62 (11.16)	0.001	0.22
Unchanged	55	110.78 (20.84)	2.32 (13.13)	0.196	0.11
Worsened	101	109.62 (22.11)	7.75 (12.13)	< 0.001	0.35
0.5 effect size					
Improved	17	98.75 (31.20)	− 12.04 (12.79)	0.001	0.39
Unchanged	136	110.14 (21.33)	1.05 (11.52)	0.288	0.05
Worsened	55	111.89 (21.53)	12.35 (11.25)	< 0.001	0.57
Using only participants with complete CRRS data *N* = 171					
0.2 effect size					
Improved	44	109.57 (26.14)	− 5.52 (11.64)	0.003	0.21
Unchanged	43	112.09 (21.33)	1.58 (11.48)	0.371	0.07
Worsened	84	107.31 (22.18)	8.18 (12.90)	< 0.001	0.37
0.5 effect size					
Improved	15	101.40 (32.38)	− 12.60 (13.53)	0.003	0.39
Unchanged	110	109.93 (22.27)	0.71 (10.89)	0.496	0.03
Worsened	46	109.61 (21.21)	13.54 (11.43)	< 0.001	0.64

^a^ $${{\left| {{\text{Mean}}\;{\text{change}}\;{\text{score}}} \right|} \mathord{\left/ {\vphantom {{\left| {{\text{Mean}}\;{\text{change}}\;{\text{score}}} \right|} {{\text{SD}}\,{\text{mean}}\;{\text{baseline}}\;{\text{score}}}}} \right. \kern-0pt} {{\text{SD}}\,{\text{mean}}\;{\text{baseline}}\;{\text{score}}}}$$

As predicted, CRRS change scores correlated significantly with change on CQOLC and WHOQOL-BREF whether using prorated CRRS scores (*N* = 209, *r* = 0.62; *N* = 208 *r* = 0.53 respectively) or only including participants with complete CRRS data (*N* = 173, *r* = 0.65; *N* = 171 *r* = 0.56 respectively).

### CRRS jobs and career scale

The Jobs and Career Scale was completed by 128, 113 and 109 caregivers at the different time points respectively. Table 6 shows baseline summary statistics for the items that make up the scale. Most of the items were skewed with respondents rating excellent Quality of Life; 2 had ceiling effects greater than 70%. Internal consistency did not reach an acceptable level at baseline (α = 0.62, corrected item-total correlation range 0.07–0.51), T2 (α = 0.58 CITC range 0.01–0.49) or T3 (α = 0.61 CITC range 0.17–0.49). Test–retest reliability was robust however: weighted Kappa ranged from 0.45 to 0.73; ICC for subscale total was 0.79 (95% CI 0.71–0.85).

**Table 6:** Baseline descriptive statistics and weighted kappa for items on the Jobs and Career Subscale (*N* = 128)

	Item	Missing data (%)	Mean ± SD	Median/mode	$\left| {{\text{z-score}}\;{\text{skew}}} \right|$	% Floor (score 0)	% Ceiling (score 4)	Inter-item correlations	CITC	Weighted κ
CE3	I have reduced my working hours because of X’s illness	0	3.31 ± 1.15	4.0/4	7.87	4.7	64.8	− 0.05 to 0.45	0.07	0.62
CE4	I have increased my working hours to compensate for loss of X’s income [reverse scored]^a^	0	3.89 ± 0.49	4.0/4	27.94	0.8	93.0	− 0.05 to 0.33	0.21	0.73
CE5	My working hours are flexible to accommodate my caregiving responsibilities (e.g. attending appointments)	0.8	2.39 ± 1.51	3.0/4	1.67	16.5	35.4	− 0.06 to 0.53	0.20	0.56
CE6	I feel I am able to do my job as well as I would like	0	2.59 ± 1.30	3.0/4	2.69	9.4	32.0	0.05–0.41	0.50	0.45
FT9	I am concerned about keeping my job and income [reverse scored]	0	3.30 ± 1.18	4.0/4	8.18	7.0	65.6	0.12–0.47	0.50	0.59
CE7	I feel that supporting X has limited my career opportunities [reverse scored]	0	3.57 ± 0.97	4.0/4	11.52	3.1	78.1	0.03–0.47	0.42	0.65
CE8	I feel supported by my employer	1.6	2.83 ± 1.30	3.0/4	4.05	8.7	42.9	− 0.15 to 0.53	0.51	0.71

^a^Where items are reverse scored, the response ‘not at all’ is given the maximum score ‘4’

## Discussion

The CRRS was developed as a self-report instrument for informal caregivers. Informed by concept mapping of current PRO content, it was developed through extensive qualitative work to address the identified neglected areas. It is a comprehensive measure providing a profile of five subscales: Emotional Health and Wellbeing, Lifestyle, Support and Impact, Self-Care and Financial Wellbeing. The standalone subscale, Jobs and Careers is completed only by participants currently in paid employment. The format and structure are compatible with the FACIT Measurement System. Developed initially with the informal caregivers of people with advanced cancer, it is nevertheless sufficiently generic for use in early stage cancer and with other chronic conditions.

Our method of recruiting caregivers for all of the PROACT studies was to have patients nominate their primary informal caregiver. Defined as ‘the person who is your main source of support’, caregivers could be providing emotional and/or practical support, such as attending appointments, not necessarily more personal care tasks. Thus some participants might have been providing emotional support every few days by telephone, while others would be engaged in personal care such as help with dressing or washing. While the cancer stage is important, so is the level of caregiving that is provided, and this varied within both early and advanced groups. For example, a patient with early stage cancer may need considerable support in many aspects of life immediately after surgery and during treatment, while someone who had surgery for grade III melanoma some years previously and is currently only having regular scans may need relatively little support. Because the questionnaires have been developed and validated in such a range of caregivers, we feel confident that the items included are relevant and important at any level of caregiving and can provide a useful measure over time if the trajectory changes. Furthermore, the very low rate of missing data in study 4 leads us to believe that the items are acceptable, appropriate and possible to answer for caregivers to patients with early stage cancer.

The CRRS appears to be acceptable to participants both in terms of time to complete and the very low missing data rate in total and per item. Missing data were notably higher for three items, which have been excluded from the EFA and any subscale scores. No participants recorded minimum or maximum scores on the final scale.

The inclusion of a total score as well as a profile of subscale scores is a topic of discussion. We believe there is utility in a total score, particularly when the scale is being used as part of a clinical discussion for straightforward monitoring of global change over time and as a way to alert both the caregiver and healthcare professional that they may be experiencing difficulties and may benefit from further discussion and/or investigation. That said, some caution may be warranted in the use of a total score as the correlation between ‘emotional health and wellbeing’ and ‘lifestyle’ subscale scores is quite high (*r* = 0.62) and there is a risk these factors might dominate the score.

The 41 core items yield a total score and 5 subscale scores. All demonstrated good internal consistency and test–retest reliability. The total score can be calculated using all 41 items or excluding the three standalone items, either score showed the predicted strength of correlation with measures for criterion and convergent validity. The scale demonstrated responsiveness to change. Whether a threshold effect size of 0.2 or a more conservative 0.5 was used, caregivers showing improvement on the anchor variable showed significant improvement in CRRS scores and those declining on the anchor variable showed significant decline in CRRS scores.

We did not make any a priori hypotheses on group differences on either the total or separate subscale scores. We found that age was a significant factor with significant post-hoc contrasts between those over 65 years and those aged 51–65, and those over 65 years and those 50 years or younger. Retired caregivers had significantly higher scores than those working and those not in paid employment. Those working had significantly higher scores than those not in paid employment. Caregivers who had given up work because of their caregiving responsibilities had significantly lower scores than those who had not (either because they were not working or had continued to work) and those caring for someone currently on treatment, significantly lower than those caring for someone not receiving treatment. There was no difference in scores for those who had additional caregiving responsibilities, such as for children or grandchildren, and those who did not, although previous research suggests that the impact for caregivers is likely to be related to the number of other social roles they fulfil [3]. Further exploration of these potential cumulative effects and the role of age and employment will be performed at a later date.

The instrument had good validity and reliability overall but there were features of the data that warrant caution. Although not displaying ceiling effects, the total score is negatively skewed. The Financial Wellbeing subscale is particularly skewed with 10% (still below our cut-off threshold) of participants having maximum score. This may reflect the fact that UK patients do not pay for medical treatment and that a large proportion of our sample were already retired so the financial implications due to loss of employment for example are less pronounced.

While internal consistency and test–retest reliability for the subscales is good, in some cases the corrected item-total and inter-item correlations are lower than 0.5 and 0.3 respectively raising the question that some items may not be contributing significantly to the subscales and the data might benefit from further item level analysis. Similarly while the test–retest reliability for the Jobs and Careers subscale and individual items was good; internal consistency for this scale was disappointing and warrants further investigation.

## Limitations and future research

We developed the measure in caregivers to people with stage III/IV cancer and in a limited number of tumour groups which although chosen to ensure a spread of key patient characteristics such as age, inevitably resulted in some sampling bias. Additionally while a majority (80.5%) of UK population are White British, 94% of our participants described themselves as this. Cultural validity needs to be established as informal caregiving and the expectations on family members are perceived differently across cultures.

We invited patients to nominate the person who they felt was their main source of support. This resulted in a limited range of relationship ‘types’ between patient and caregiver; 78% of caregivers were the partner or spouse of the patient. This means we are unable to make meaningful comparisons between different types of relationship, but reflects the pattern of support in this group of patients.

In this study we measured change in PRO scores over time but have not explicitly linked these to marked changes in trajectory such as transition to palliative care or from diagnosis through treatment. This is an avenue for future research as caregiving responsibilities are likely to vary in line with different lines of treatment [9]. Furthermore, Girgis et al. [27] report that caregiver unmet needs shift from cancer care-related needs towards their own needs with increasing time since diagnosis. In the current study there were no differences in CRRS *total* score by time since diagnosis (less than a year, 1–2 years or more than 2 years), however a more detailed analysis of responses to items which are more cancer-care centred versus those which are more caregiver centred may be valuable to explore any difference over time.

The CRRS is comprehensive. Future work will examine which items are most descriptive of the underlying dimensions. Using IRT methods we will investigate the possibility of reducing the number of items or producing useful short forms.

## Conclusion

The importance of considering the needs of caregivers as well as patients is recognised for the provision of good care [28, 29].The use of PROs for caregivers alongside those for patients might facilitate discussions about potential treatments and supportive interventions needed for both. There has been an increase in the development of services for caregivers, [30, 31] now there is need for robust measures to quantify the effectiveness of any such intervention. The CRRS and its subscale scores may provide useful indications as to how different domains are impacted for different individuals, allowing for a more tailored approach to supportive interventions [32]. This initial validation suggests that the CRRS shows promise. Further work, including item level analysis is being conducted.

## Electronic supplementary material

Below is the link to the electronic supplementary material.

Supplementary material 1 (DOCX 14 KB)
